# Genomic Insights into Local Adaptation and Evolutionary Trajectories of *Propylea japonica*

**DOI:** 10.3390/biom16030421

**Published:** 2026-03-12

**Authors:** Lijuan Zhang, Yan Shi, Mengqi Wang, Yang Xu, Xiaojie Yang, Man Zhao, Weizheng Li, Xianru Guo, Chenchen Zhao, Yuqiang Xi

**Affiliations:** 1 College of Plant Protection, Henan Agricultural University, Zhengzhou 450046, China; zhanglijuan@henau.edu.cn (L.Z.);; 2 Society Work Department of the CPC Suifenhe Municipal Committee, Suifenhe 157399, China

**Keywords:** adaptive evolution, biological control agent, evolutionary history, genetic divergence, population expansion

## Abstract

As an effective biological control agent, *Propylea japonica* (Coleoptera: Coccinellidae) preys on aphids, whiteflies, planthoppers, and small caterpillars, playing a crucial role in pest management within agro-ecological systems. However, the lack of population genomic data has hindered efforts to optimize its use in biological control. We anayzed resequencing data from 166 genomes across 29 populations spanning *P. japonica*’s distribution in China. This study reconstructed the species’ evolutionary history, assessed population genetic diversity and demographic structure and identified the key environmental factors driving adaptive evolution. Meanwhile, we predicted its suitable habitats across different periods using ecological niche modelling methods. The results indicated that North China (G1, Yellow River Basin) was the likely geographic origin of *P. japonica*. Northern and southern populations show significant genetic differentiation, with adaptive evolution in the south being the major driver. We identified genomic signatures of selection in adaptive genes associated with increased pesticide resistance and thermal tolerance. Over the past 20,000 years, effective population size of *P. japonica* experienced an early bottleneck during the Last Glacial Maximum period, and a subsequent rapid expansion. These insights are critical for improving the conservation and application of natural enemies, ultimately enhancing biological control in agricultural systems.

## 1. Introduction

The *Propylea japonica* (Coleoptera: Coccinellidae) is a key predator in agroecosystems, preying primarily on aphids, whiteflies, planthoppers, and small caterpillars [1]. Native to China, this species is widely distributed and exhibits high genetic and phenotypic diversity. It has also demonstrated strong resilience to environmental stressors, including high temperatures and heavy pesticide use [2,3], making it a valuable agent for biological control. The extent to which its adaptations are shaped by evolutionary history depends largely on the selection pressures acting on its phenotype. Understanding the evolutionary trajectory and environmental adaptation of *P. japonica* using genomic data is essential for driving the transition of biological control from empirical and reactive approaches to predictive, precision-oriented, and preventative management. Particularly for genetically differentiated populations, comprehensive population genomic investigations can provide early molecular insights for the precise zonal protection of predators.

Environmental tolerance and adaptation are crucial for insect evolution, shaping fitness traits at both individual and population levels. These processes drive changes in genetic diversity, population structure, and the formation of evolutionarily significant units. However, the genetic basis underlying these adaptations remains complex. With rapid technological advances, we can now integrate ecological niche modelling (ENM) with genome sequencing data (WGR) to project future habitat changes while also characterizing the adaptive genetic diversity within populations. These integrative approaches can address the identification of adaptive traits, the vulnerability of populations to environmental shifts, and the geographic patterns of local adaptation [4,5,6].

Predatory insects occupying higher trophic levels, are particularly vulnerable to environmental shifts, making them prone to demographic fluctuations and local extinctions. Some natural enemies have evolved adaptive traits, including insecticide resistance [7] and thermal tolerance [8], which enhance their ability to cope with environmental stress. Intraspecific variation regarding resistance has been extensively documented in insects. Genomic analyses suggest that highly resistant populations exhibit stronger signatures of adaptation across their genomes. However, the extent to which these environmental stressors shape population differentiation and how evolutionarily significant units respond to these pressures remain unclear. Investigating the genomic basis of adaptation in *P. japonica* is crucial for elucidating the molecular mechanisms underlying its response to climate change and pesticide exposure. Such insights will help predict how different populations may respond to future environmental changes and inform strategies for integrated pest management (IPM). By increasing the abundance of superior populations, these strategies could enhance the role of biological control in agricultural systems.

In this study, individual samples originating from wild populations across China underwent genomic analysis. This research focuses on three key aspects: (1) elucidating the formation of population genetic patterns, including population origins, genetic structure, genetic diversity, inter-population relationships, and the evolutionary history of *P. japonica*; (2) examining the feedback mechanisms between population genetic evolution, pesticide usage, and temperature-driven selection; and (3) uncovering the molecular mechanisms underlying population genetic differentiation in response to increasing temperatures and pesticide exposure in *P. japonica*.

## 2. Materials and Methods

### 2.1. Sample Collection and DNA Extraction

**Figure 1:**
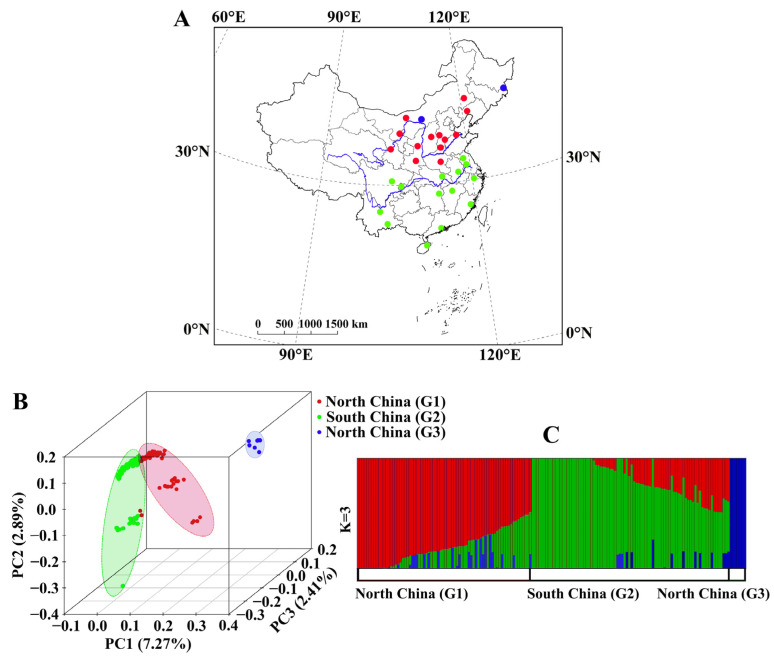
Population sampling and genetic structure. (**A**) geographic distribution of samples, (**B**) PCA plots, and (**C**) ADMIXTURE analysis for K = 3.

A total of 166 *P. japonica* individuals were collected from 29 locations across China (Figure 1A; Table S1). Sampling efforts aimed to capture diverse eco-climatic conditions and varying generational cycles. Most locations provided six to eight individuals, except for Bayannur (3 individuals) and Chifeng (2 individuals) in Inner Mongolia; Dongning, Heilongjiang (3 individuals); Nangong, Hebei (2 individuals); and Jinzhou, Liaoning (3 individuals). Specimens were immediately preserved in 95% ethanol and stored at −80 °C prior to DNA extraction. To minimize DNA contamination, midguts were removed before extraction. Genomic DNA was isolated using a DNeasy Blood and Tissue Kit (Qiagen, Hilden, Germany), following the manufacturer’s protocol.

### 2.2. Genome Sequencing, Data Mapping, and SNP Calling

Genomic DNA was sequenced using the Illumina HiSeq 2000 platform, generating 150 bp paired-end reads. The majority of samples (*n* = 164) were sequenced at 5–10× coverage, while two were sequenced at ~20× coverage, a strategy commonly employed in insect genomics [9,10]. In total, 16,376,014 raw reads were generated. Reads with >10% ambiguous bases (‘N’), >50% low-quality bases (Q < 10), adapter contamination, or identical sequences at both ends were discarded. The Burrows-Wheeler Aligner (BWA 0.7.17) [11] was used to map high-quality reads (16,352,670) onto the *P. japonica* reference genome [12]. Mapping efficiency was assessed by calculating the ratio of clean reads to total reads, and paired-end alignments were verified using samtools. Unpaired alignments were removed using default parameters, except for the “-no-mixed” option).

Duplicate reads were marked and removed using Picard-tools http://sourceforge.net/projects/picard/ (accessed on 2 March 2026). Properly paired and collapsed reads were retained. Variant calling was performed with the Genome Analysis Toolkit (GATK 4.0). Haplotype Caller [13] using the parameters --emitRefConfidence GVCF --variant_index_type LINEAR --variant_index_parameter 128,000. Small insertions and deletions were identified by comparing 166 genomes against the reference genome. Each individual was initially processed to generate a gVCF (Variant Call Format) file, followed by joint-genotyping to produce a final multi-sample VCF file. Variants were filtered using the following criteria: (1) SNPs within 5 bp of an indel and adjacent indels within 10 bp were removed using vcfutils.pl in bcftools (varFilter-w 5-W 10); (2) No more than two variations were allowed within a 5 bp window (clusterSize 2, clusterWindowSize 5); (3) SNPs with QUAL < 30.0, QD < 20.0, MQ < 40.0, or FS > 60.0 were excluded; (4) Additional filtering was performed using GATK’s default parameters. Only SNPs passing quality control were retained for downstream analyses.

SNP annotation was performed using SnpEff [14] based on the *P. japonica* reference genome. Variants were classified into intronic, exonic, coding sequence, intergenic, upstream, downstream, and splicing regions, with functional predictions for synonymous and non-synonymous mutations.

### 2.3. Phylogenetic Analysis and Genetic Structure

A total of 27.25 million high-quality SNPs in 166 individuals were retained for phylogenetic analysis. A neighbor-joining (NJ) tree was constructed using MEGA X [15] under the Kimura 2-parameter model with 1000 bootstrap replicates. To resolve taxonomic relationships among subpopulations and infer evolutionary timelines, both rooted and unrooted trees were generated, with *Harmonia axyridis* serving as the outgroup for the rooted tree. In addition, the maximum likelihood (ML) approach was employed to conduct mutual validation with the NJ method, thereby testing the robustness of the results. The ML method was executed using IQ-TREE 2.2.0 software under the maximum likelihood criterion; the substitution model was set to MFP, with the default ultrafast bootstrap approximation involving 1000 replicates.

Population structure was examined via Principal Component Analysis (PCA) using EIGENSOFT 7.2.1 [16] under default settings. Additionally, genetic clustering was assessed with ADMIXTURE 1.22 [17], testing values of K from 1 to 10. The optimal K was determined based on the lowest cross-validation error. Linkage disequilibrium (LD) was evaluated using Plink 2.0 [18]. The r^2^ value was calculated for each chromosome using SNPs with the following parameters: –ld-window-r^2^ 0–ld-window 999,999–ld-window-kb 1000. Then, genome-wide averages were computed. Genetic differentiation (Fst) between populations was estimated in MEGA X using sites with >80% coverage, applying the Kimura 2-parameter model with 1000 bootstrap replicates.

To assess the correlation between genetic and geographic distance, the vegan R package were used. The analysis included 29 populations, excluding an outlier from Honghe, Yunnan (YNH). A matrix of pairwise Fst values, derived from 158 nucleotide sequences spanning 39,747 sites, was used for correlation analyses. Geographic distances between populations were calculated based on latitude and longitude.

### 2.4. Ecological Niche Analysis of P. japonica

We used the maximum entropy model (Maxent) to analyze the ecological niche of *P. japonica*. Occurrence data were obtained from the Global Biodiversity Information Facility (GBIF), available online: https://doi.org/10.15468/dl.zs6e22 (accessed on 1 March 2026) and supplemented with our collection records and literature data. To minimize spatial autocorrelation, we filtered the occurrence points using the ENMTools R package [19], resulting in 104 unique localities for analysis. Nineteen bioclimatic variables covering the present (1970–2000), Last Glacial Maximum (LGM), and mid-Holocene periods at a 5-arc-minute resolution (Community Climate System Model, CCSM4) were downloaded from WorldClim. Available online: https://doi.org/10.1002/joc.5086 (accessed on 1 March 2026). Highly correlated variables (Pearson’s correlation coefficient *r* > 0.75) were excluded, leaving six: precipitation of the warmest quarter (BIO18), mean temperature of the wettest quarter (BIO8), isothermality (BIO3), annual mean temperature (BIO1), temperature seasonality (BIO4), and mean temperature of the warmest quarter (BIO10). To predict future distributions, we retrieved climate projections at 5-arc-minute resolution under three Shared Socioeconomic Pathway (SSP) scenarios [SSP1-2.6 (low forcing), SSP3-7.0 (medium-to-high forcing), and SSP5-8.5 (high forcing)] from the CMIP6 archive for four future periods: 2021–2040, 2041–2060, 2061–2080, and 2081–2100. We optimized model parameters by testing 29 combinations of five feature classes and 40 regularization multipliers, selecting the model with the lowest Akaike Information Criterion corrected (AICc) value. Model performance was evaluated using the area under the curve (AUC). Suitable habitat projections were generated by applying the current climatic requirements of *P. japonica* to past (LGM) and future (CMIP6) climate scenarios. Each analysis was replicated using cross-validation. ArcGIS 10.x was used to convert Maxent outputs into binary distribution maps, resulting in present-day range maps and future projections.

### 2.5. Evolutionary History

To reconstruct the demographic history of *P. japonica*, the Pairwise Sequentially Markovian Coalescent (PSMC) model [20] was implemented with default parameters to infer historical changes in effective population size. PSMC employs hidden Markov models (HMMs) to identify historical recombination events and estimate the time to coalescence between homologous chromosomes. This approach has been widely used to infer population fluctuations and complement demographic reconstructions [21]. Consensus sequences were generated using samtools, with bases masked if their depth was less than one-third or more than twice the mean coverage. Two representative samples, one from northern China (G1) and one from southern China (G2), with genome coverage exceeding 20×, were analyzed using PSMC 0.6.5 with parameters-N25-t15-r5-p ‘4 + 25*2 + 4 + 6’. To assess variance in estimates, the 100 bootstrap replicates were conducted.

For higher resolution of recent demographic history, SMC++ 1.0 was used, which has demonstrated superior performance for recovering population dynamics over short timescales [22]. Additionally, the generalized phylogenetic coalescent sampler (G-PhoCS 1.2.3) was implemented to infer ancestral population sizes and migration rates. G-PhoCS estimates parameters through a Bayesian framework, combining Markov Chain Monte Carlo (MCMC) sampling of genealogies and model parameters. Each MCMC chain was run for 2,000,000 generations, sampling every 20th iteration. Convergence and burn-in were evaluated using TRACER 1.7 [23]. A mutation rate of 8.4 × 10^−9^ per base per year—based on *Drosophila* [24] estimates and a generation time of 0.14 years (derived from field observations and literature)—was applied to PSMC 0.6.5, SMC++ 1.0, and G-PhoCS 1.2.3 analyses.

### 2.6. Detection of Adaptive Signals

The PopGenome package in R was used to calculate genome-wide genetic diversity parameters within 100 kb sliding windows (10 kb step size), including nucleotide diversity (*θ*_π_), Watterson’s estimator (*θ*_w_), population differentiation (Fst), Tajima’s *D*, and Fu and Li’s *F* and *D* statistics. The SNP dataset was filtered for a minor allele frequency (MAF) > 0.05 and integrity ratio > 0.5, with windows containing fewer than 10 SNPs excluded. Outlier windows were identified based on the empirical 5% (or 1%) threshold for *θ*_π_ ratio and Fst. Selection scans were carried out by comparing G1 to G2 and G3 (G1/G2, G1/G3, G2/G3) and reversely (G2/G1, G3/G1, G3/G2). Given that G1 exhibited higher *θ*_π_ and *θ*_w_ values and occupied a basal position in the phylogenetic tree, these results of G1/G2, G1/G3 and G2/G3 were the primary focus. Windows were considered significant if Tajima’s *D* was below the 95% confidence limit for the corresponding population size. Fst and *θ*_π_ ratios were visualized using R scripts. Candidate genes identified in selective sweeps were functionally annotated using Blast2GO v2.5.0 [25]. Pathway enrichment analysis was performed using the KEGG database, available online: https://www.genome.jp/kegg/pathway.html (accessed on 2 March 2026), with additional annotation through the NCBI non-redundant protein (NR) and KOG databases.

## 3. Results

### 3.1. Assembly and Genomic Variation

All individuals were sequenced using whole-genome sequencing, generating 1.11 Tb of clean data. After quality filtering, 3.71 Gb of clean reads (approximately 92.48% of total reads) were mapped to the *P. japonica* genome [12]. Individuals with a mapping rate or coverage below 60% were excluded (Table S2). The final genomic dataset comprised 27,249,260 SNPs and 2,944,789 indels with an MAF ≥ 0.05. Among these, 394,357 SNPs were functionally significant, causing start codon losses or stop codon gains or losses (Tables S3 and S4).

### 3.2. Phylogenetic Evolution and Genetic Diversity

To assess genetic relationships, principal component analysis (PCA), admixture analysis, genetic differentiation (Fst), correlation analysis between genetic and geographic distances, and phylogenetic tree reconstruction were used. The PCA revealed three major groups, explaining ~12.57% of the total genetic variation (Figure 1B). The group G3, comprising individuals from Baotou, Inner Mongolia (NMGB, six individuals) and Dongning, Heilongjiang (HLJ, one individual), was distinctly separated from the rest. The G1 and G2 groups were also differentiated, though with some admixture. G1 encompassed populations from both sides and north of the Yellow River, while G2 included populations along and south of the Yangtze River. This geographic structuring was further supported by maximum likelihood-based clustering analysis (Figure 1C). At K = 2, G3 was the first to separate, indicating a distinct genetic composition. At K = 3, the optimal K-value (Figure S1), the genetic ancestry mainly from both groups of G1 and G2 and the asymmetric genetic admixture events seem to exist between G1 and G3, and G1 and G2 regional groups. Genomic differentiation analysis showed low divergence between G1 and G2 (Fst = 0.026), consistent with recent divergence and ongoing gene flow. However, significant genetic differentiation was observed between G1 and G3 (Fst = 0.367) and between G2 and G3 (Fst = 0.388).

These results highlight clear genomic distinctions between northern and southern populations. Among individual populations, Baotou (G3) exhibited the highest differentiation (Fst > 0.35, except for HLJ, where Fst = 0.20), followed by Heilongjiang (HLJ, Fst > 0.10).

A significant positive correlation between genetic and geographic distance was detected at the whole-genome level, as supported by vegan (r = 0.38, *p* = 0.019) analyses, suggesting isolation by distance as a key driver of genetic variation.

The neighbor-joining (NJ) tree (Figure S2A) revealed that G1 clustered with some individuals from G3, suggesting a shared ancestry. Within G1, two large clades were identified. G2 formed a major clade with four smaller subclades, two of which were closely related to G1, consistent with PCA results (Figure 1B). Notably, individuals from transitional regions (e.g., Sichuan, Chongqing, Jiangsu, and Hubei) were found within these intermediate clades. 

**Figure 2:**
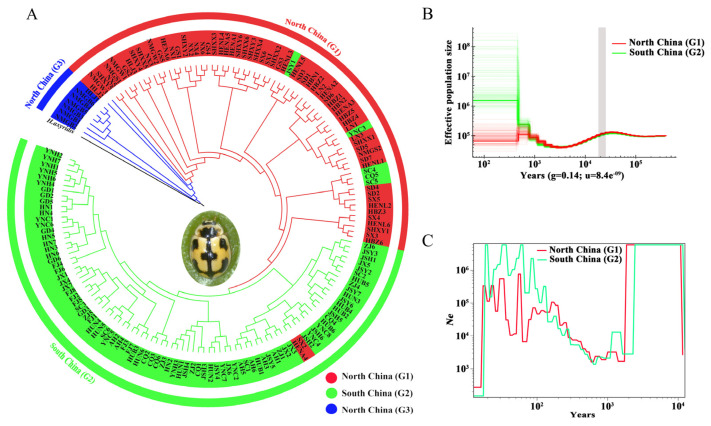
Phylogeny and demographic history. (**A**) ML phylogenetic tree, (**B**) Demographic history of *Propylea japonica* inferred from the PSMC model. The grey shading indicates the period of LGM, and (**C**) SMC++-based reconstruction of the demographic history.

When *H. axyridis* was used as an outgroup (Figure 2A and Figure S2B), populations from North China (G1) and G3 were positioned as basal lineages closest to *H. axyridis*, with G3 being the most proximate to the outgroup. This suggests that the G3 lineage may represent an early-diverging or incipiently differentiated group. The clustering patterns in Figure 2A were consistent with those in Figure S2, with most individuals from the same regional groups clustering together. Although in the ML tree, five individuals from G2 were grouped with G1, two G1 individuals appeared in the G2 clade. In the NJ tree, nine individuals from G2 were grouped with G1, while only one individual from G1 was assigned to the G2 lineage. Individuals from the southernmost populations (Honghe, Yunnan; Chuxiong, Yunnan; Zhuhai, Guangdong; Haikou, Hainan) clustered at the tree’s apex, reflecting greater divergence. Collectively, these findings support the hypothesis that the northern group (G1) represents the ancestral lineage from which both G3 (northern divergent group) and G2 (southern group) emerged.

As shown in Table S5, genetic diversity parameters, including the number of segregating sites (n), nucleotide diversity (*θ*_π_), and Watterson’s estimator (*θ*_w_), exhibited a decreasing trend from G1 to G3, with G1 displaying the highest genetic diversity. Compared to pest species, *P. japonica* exhibited relatively low genetic diversity. This reduction may be attributed to the higher sensitivity of predators to environmental changes due to their specialized diet. Additionally, population expansion (as detailed in the demographic history analysis) may have initially reduced genetic diversity at the expansion front, ultimately leading to lower overall genetic variation. Despite this, *P. japonica* exhibited large heterozygous peaks in k-mer distribution, with a heterozygosity rate of ~0.9% [12]. Genome-wide heterozygosity rates were highest in G1 (34.15%) and G2 (33.69%), while G3 was comparatively lower (24.50%). The observed low allelic diversity alongside high heterozygosity may be explained by rapid range expansions driven by dispersal. High dispersal capacity of *P. japonica* can preserve heterozygosity during expansion. Moreover, allelic diversity typically declines more rapidly than heterozygosity in expanding populations.

The reference genome contained, on average, one variant every 28 bp, with an estimated ~1 heterozygous site per 100 bp. Linkage disequilibrium (LD) analysis revealed the highest LD in G3, followed by G1 and G2 (Figure S3), suggesting stronger selection pressure in G3. The ratio of non-synonymous to synonymous substitutions was comparable across the groups (G1 = 0.90, G2 = 0.90, G3 = 0.92).

### 3.3. Population Demographic History

PSMC analysis was used to reconstruct long-term effective population size (*Ne*) trajectories. The results revealed similar demographic histories in North and South China, characterized by one decline and two expansions over the last 100,000 years (Figure 2B). The first expansion occurred ~25,000–70,000 years ago, followed by a decline during the Last Glacial Maximum (LGM, ~20,000 years ago), reaching a low point ~2000–7000 years ago. After a subsequent increase in population size, the divergence between G1 and G2 began to emerge in the recent hundreds with low resolution. This pattern aligns with findings from Wang et al. (2023) [26], which indicate that *P. japonica* expanded after experiencing a bottleneck.

SMC++ analysis estimated divergence times and *Ne* trends in the recent past. When considering only G1 and G2, their *Ne* curves diverged ~25–400 years ago (Figure 2C), with G2 maintaining a higher *Ne* during divergence, consistent with the PSMC results. However, when all three groups were analyzed, their *Ne* curves remained indistinguishable, with population expansion occurring at a rate ~10^3^ times higher than in the recent past (~1000 years).

PhoCS analysis estimated the divergence of G1 and G2 at ~28.3 million years ago [95% confidence interval (CI): 24.0–32.3 million years], with a 13.08% increase in ancestral *Ne*. The divergence of G3 from G1 and G2 was estimated at ~191.1 million years ago (95% CI: 131.1–240.2 million years), accompanied by a 48.73% reduction in *Ne*. Gene flow analysis indicated ongoing exchange among the three geographic regions, with higher levels of gene flow from G3 to G1/G2 than in the reverse direction (Table S6).

Neutrality tests (Tajima’s *D*, Fu and Li’s F*, and Fu and Li’s D*) were performed to assess deviations from mutation-drift equilibrium. These parameters were positive across populations and groups, except in HLJ and LN (Tajima’s *D*, −0.959 and −0.190, respectively). G3 exhibited consistently lower values than G1 and G2, while G2 had higher values than G1. This positive distribution was consistent with patterns observed in *Hyphantria cunea*, suggesting that the recent expansion of *P. japonica* in China has not yet fully manifested in its genetic signature.

**Figure 3:**
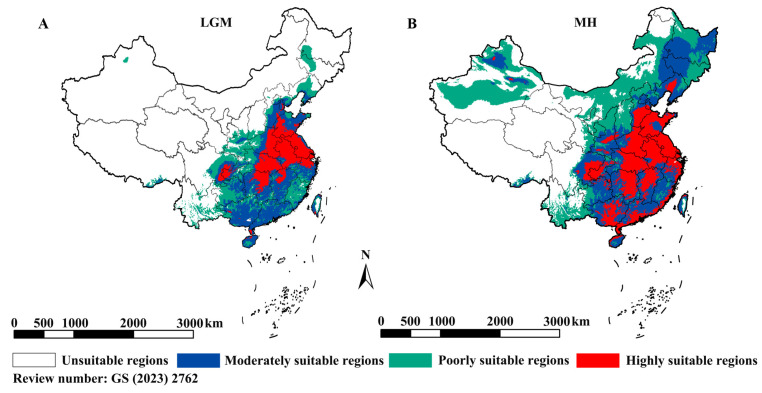
Distribution of suitable regions. (**A**) LGM climate condition; (**B**) MH climate condition.

To assess habitat suitability, climate models were constructed, yielding a high mean AUC (0.888). The niche model, based on present-day climate conditions, indicated that highly suitable habitats were primarily located in northern China, with fragmented distributions in the south. Unsuitable areas included western, northeastern, and some southernmost regions [27]. Simulations under past climatic conditions suggested that suitable habitats contracted during the LGM, with high suitability persisting only in eastern coastal and select northern regions (Figure 3A). By the mid-Holocene (~7500–3000 years ago), highly suitable areas expanded southward (Figure 3B). Future projections (2081–2100) predict further expansion into northern, northeastern, and western China, particularly under high-emission scenarios. Overall, these results suggest that *P. japonica* experienced a bottleneck, differentiated within China, and dispersed across most of the country in response to climate warming.

### 3.4. Genomic Signatures of Local Adaption

The genomic regions subject to selection were identified based on high *θ*_π_ ratios in G1/G2, G1/G3, and G2/G3 comparisons, along with extreme Fst values reflecting allele frequency divergence. The 102 selective-sweep windows (average size: 617.65 kb; total ~63 Mb) with Fst values > 0.237 and *θ*_π_ ratios > 1.816 in G1/G2 were found by using a combination of *θ*_π_ ratios and Fst values. Similarly, 31 selective-sweep windows (average size: 370.97 kb; total ~11.5 Mb) were identified in G1/G3 (Fst > 0.678, *θ*_π_ > 11.301), and 24 selective-sweep windows (average size: 259.60 kb; total ~6.23 Mb) were found in G2/G3 (Fst > 0.712, *θ*_π_ > 11.124). These results highlight significant differentiation in localized genomic regions among the groups.

**Figure 4:**
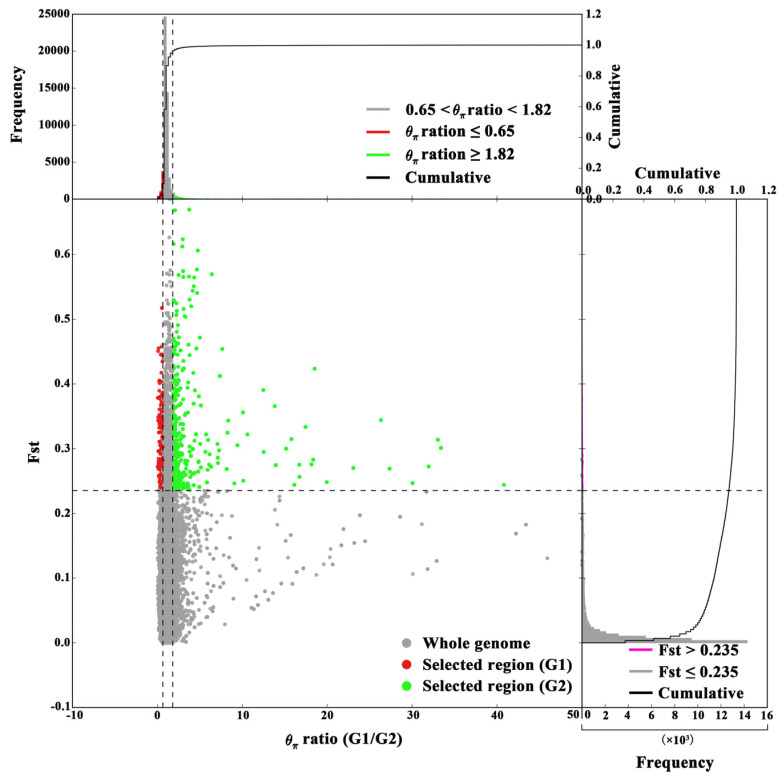
Selection signatures between G1 and G2 lineages. Distribution and frequency of *θ*π ratios (X axis) and Fst (Y axis). Grey horizontal dotted lines indicate their highest 5% thresholds. Green dots indicate genomic regions under selection for G2 lineage and red dots for the G1 lineage.

**Figure 5:**
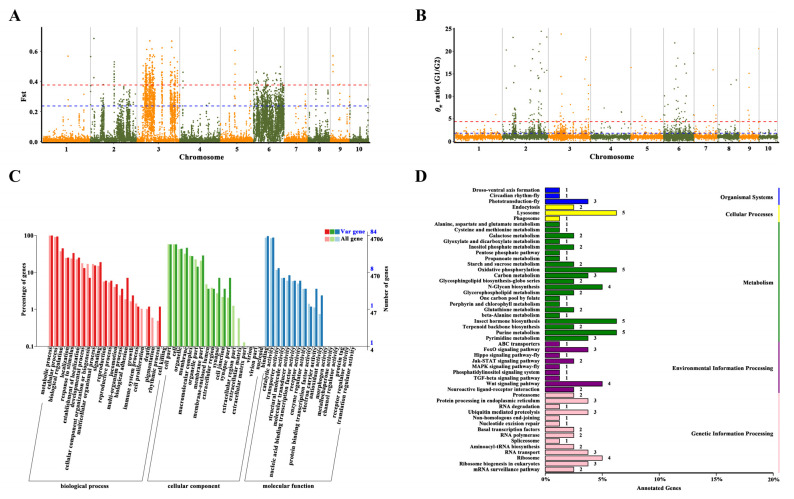
Selection signatures and functional enrichment of selected genes between G1 and G2 lineages (G1/G2). The Fst (**A**) and *θ*π (**B**) ratios values around selection signatures, blue horizontal dotted lines indicate their highest 5% thresholds, red horizontal dotted lines indicate their highest 1% thresholds, (**C**) Functional enrichment of genes based on GO classification, (**D**) Functional enrichment analysis of annotated genes from KEGG database. The numerical labels on the right side of the bar chart indicate the gene count.

Functional enrichment analysis for G1/G2 (top 1% threshold) revealed that candidate genes were predominantly associated with molecular functions such as metal ion binding (GO:0046872) and hydrolase activity targeting carbon–nitrogen (but not peptide) bonds (GO:0016810), as well as biological processes including larval behavior (GO:0030537), larval locomotory behavior (GO:0008345), and metabolism (GO:0008152) (Table S7). Expanding the threshold to the top 5% (Figure 4 and Figure 5A,B; Table S8) uncovered additional molecular functions, including heme binding, phosphatase regulator activity (GO:0019208), and antioxidant activity (GO:0016209). In *Rhodnius prolixus*, a blood-feeding insect, silencing a heme-binding protein disrupts embryonic mitochondrial function and impairs development [28]. In *Ostrinia furnacalis*, aliphatic nitrilase expression increases under cold acclimation [29].

Biological processes associated with selection include DNA recombination (GO:0006310), larval behavior (GO:0030537), cGMP metabolism (GO:0046068), cGMP biosynthesis (GO:0006182), signal transduction via phosphorylation (GO:0023014), cyclic nucleotide metabolism (GO:0009190, GO:0009187), mesoderm development (GO:0007498), and carbohydrate derivative metabolism (GO:1901135) (Figure 5C). DNA recombination serves as a versatile repair mechanism for internal and external DNA damage and is a major driver of adaptive evolution in insects, where increased recombination rates mitigate age-related oxidative stress [30] and influence population survival under heat stress [31].

cGMP, a key cellular second messenger, plays a crucial role in stress response. Its downstream effector, cGMP-dependent protein kinase (PKG), phosphorylates specific substrates to activate the cGMP/PKG pathway. In *Drosophila melanogaster* and *Locusta migratoria*, PKG mediates synaptic and central circuit responses to thermal stress [32], and regulates larval foraging behavior across invertebrate species, including *D. melanogaster* and *Apis mellifera* [33].

Mesoderm development is essential for organogenesis, influenced by genetic and epigenetic variations under selective pressures such as environmental changes. In *Drosophila*, deleting key transcription factors (Nrf orthologs, CncA/B/C) essential for mesoderm development leads to embryonic arrest and impaired oxidative stress mitigation [34].

In conclusion, analysis of the South China group, incorporating additional databases (KEGG, Figure 5D; KOG, Figure S4), revealed enrichment in pathways related to larval behavior, signal transduction, energy metabolism, environmental adaptation, and stress tolerance, indicating key mechanisms underlying local adaptation.

The several genes were identified in the South China lineage (Table S9), which include two cytochrome P450 monooxygenase (CYP450) genes, *Synaptotagmin 1* isoform X1 (*SYT1*), kinesin-like protein subito isoform X2 (*TPX2*), chitin deacetylase 4 precursor (*CDA4*), and *Zinc finger protein 1*, transcript variant X4 (*ZFP4*). CYP450s are involved in drug metabolism, biotransformation of natural compounds, and the oxidative metabolism of xenobiotics [35] and have been linked to pesticide resistance in *P. japonica*. SYT1, a Ca^2+^ sensor essential to neurotransmission, is also associated with feeding behavior traits related to local adaptation. Chitin deacetylases (CDAs) play a crucial role in insect growth and development and serve as potential insecticide targets, as demonstrated in *Holotrichia parallela* [36]. Zinc finger proteins are key regulators of immune responses, influencing cytokine production, immune cell activation, and immune homeostasis [37].

These genes contribute to detoxification, neural activity regulation, feeding behavior, and local adaptation. The strong selection signatures observed align with expected behavioral changes, physiological modifications, enhanced detoxification metabolism, and increased energy expenditure, facilitating the South China lineage’s adaptation to intensive pesticide exposure and frequent heat stress.

For the G3 lineage (G1/G3), the top 5% threshold (Table S10) revealed 132 genes located within selective regions (Figures S5 and S6), with seven significantly enriched GO categories, including zinc ion binding (GO:0008270, four genes, KS = 0.0229), transition metal ion binding (GO:0046914, four genes, KS = 0.0097), hydrolase activity acting on ester bonds (GO:0016788, three genes, KS = 0.0238), the MAPK cascade (GO:0000165, two genes, KS = 0.0034), and single-organism membrane organization (GO:0044802). Zinc plays diverse physiological roles in *Drosophila*, including organ development, oocyte maturation, and embryogenesis, and functions as a structural, catalytic, and signaling component in numerous enzymes and transcription factors [38]. The MAPK cascade, a fundamental signal transduction pathway, regulates stress and immune responses in eukaryotes [39], modulating cellular signaling in response to biological and environmental stimuli [40].

In the G2/G3 comparison (top 5% threshold), 98 genes were identified in selective regions (Table S11), with five significantly enriched GO categories related to molecular function: NAD binding (GO:0051287), transition metal ion binding (GO:0046914), zinc ion binding (GO:0008270), antioxidant activity (GO:0016209), and guanyl-nucleotide exchange factor activity (GO:0005085). Eleven GO categories were enriched in biological processes, including pyrimidine nucleoside monophosphate biosynthesis (GO:0009130), single-organism membrane organization (GO:0044802), and protein localization to membranes (GO:0090150) (Table S11). NAD is essential for energy metabolism, aging, cell death, and cellular function, and it directly regulates protein–protein interactions involved in DNA damage responses [41]. The establishment of protein localization to membranes is essential for regulating protein activity, as membrane proteins mediate ion transport, environmental sensing, and enzymatic functions, which are fundamental to cellular processes.

In conclusion, these genomic outliers play critical roles in stress and immune responses, mediating adaptation to biotic and abiotic stressors and activating stress-related signaling pathways. A substantial number of stress-associated genes were highly expressed in the G3 populations, suggesting acute selection pressures in response to extreme climatic events, including persistent heat waves, cold stress, and agricultural multi-hazard meteorological disasters characteristic of the North China region (G3).

## 4. Discussion

### 4.1. The Geographical Origin and Phylogenetic Relationship of P. japonica

To determine the geographical origin of the ancestral *P. japonica* population in China, all the populations were analyzed using classical indicators, which include the highest genomic diversity across various mutation types and the serial founder model of population relationships.

Based on heterozygosity ratio (het-ratio), *π*, and *n* calculations, genetic diversity was highest in the Yellow River basin (North China, G1: het-ratio = 34.15%; *π* = 4.17 × 10^−5^; *n* = 123,993), followed by the Yangtze River basin (South China, G2: het-ratio = 33.69%; *π* = 4.09 × 10^−5^; *n* = 123,088), and lowest in the North China region (G3: het-ratio = 24.50%; *π* = 1.59 × 10^−5^; *n* = 36,357). Shared SNP analysis revealed that G3 exhibited the highest number of shared SNPs across geographical populations (24,417), whereas G2 (542) and G1 (496) had significantly fewer. The increased genetic diversity and reduced between-population gene identity in the Yellow River region align with the expected evolutionary dynamics of source populations.

The ML and NJ phylogenetic trees with *H. axyridis* as the outgroup revealed that G1 and G3 form a cluster positioned closest to the outgroup. Within G1, northwestern populations were at the base, northern populations occupied an intermediate position, and a mixed subclade from the G1-G2 junction was at the top. In the G2 clade, southern populations were adjacent to the mixed subclade, with the southernmost populations positioned at the top. The phylogenetic results align with a serial founder model, confirming that *P. japonica* originated in central China (G1, Yellow River basin). Identifying the geographic origin provides critical insights that inform precision-guided introduction, help define priority conservation zones, and enhance the stability of biological control efficacy.

The G3 lineage, inhabiting the northernmost and northeastern regions of China, displayed unique evolutionary ancestry and local adaptation, consistent with the “climatic variability hypothesis” [42]. This hypothesis predicts that populations exposed to extreme environmental conditions exhibit stronger adaptive divergence and reduced variation in climate-related genes compared to those in more stable environments.

The G3 region frequently experiences harsh climatic conditions, including cold polar air invasions, extreme temperature fluctuations, and frequent meteorological disasters such as persistent cold spells (January–February, December), prolonged heatwaves (June–July), strong winds, sandstorms, and cold waves. These environmental challenges, combined with edge population effects, make the G3 lineage more vulnerable to climate shifts. The observed low polymorphism and high genetic divergence in G3 further support this hypothesis.

### 4.2. Population Historical Fluctuations

Regarding *P. japonica*’s demographic history, both G1 and G2 populations exhibited a decline in effective population size, which likely resulted from harsh climatic conditions during the Quaternary glaciations, particularly the Last Glacial Maximum (LGM), as well as vegetation changes and anthropogenic influences during the Holocene. *P. japonica* thrives in mild, humid environments, and is a typical temperate species. While East Asia experienced significant climatic fluctuations during the Quaternary, it did not undergo massive glaciation. However, the pronounced cooling and aridification during the LGM (~21,000–17,000 years ago) [43] likely led to ecological upheavals, reducing *P. japonica*’s effective population size. Similar patterns of contraction have been observed in other temperate species, including *Aquarius paludum*, *Lymantria dispar*, *Argiope bruennichi*, and *Daphnia magna*, all of which exhibited population declines below 50° N during the LGM [44].

Over the past 1000 years, effective population sizes in the Yellow River and Yangtze River basins have expanded. Species typically survive climate shifts through migration, phenotypic plasticity, or adaptive evolution, and *P. japonica* demonstrates the capacity to employ all three strategies. First, moderate gene flow between populations has been detected through molecular and ecological niche modeling, serving as a balancing mechanism between selection and genetic homogenization. Similar findings have been reported in pelagic seabirds and *Calypte anna* (Anna’s hummingbird), where gene flow enhances genetic diversity and fitness [45]. Secondly, *P. japonica* has high plasticity because diverse phenotypes have been observed in the same hosts, habitats, or different geographic regions. The variable phenotypes show differences in body size and prey capacity [46]. Warmer temperatures further increase *P. japonica*’s activity levels, enhancing its competitive interactions with other predators [3]. Third, *P. japonica* possesses a substantial number of heat-resistant genes [12], facilitating adaptation to future warming climates. Warmer periods have historically promoted population expansion and range extension, supported by demographic history and niche model predictions. Additionally, numerous candidate adaptive genes have been identified in South China populations. In previous studies, we primarily focused on biotic factors, considering tri-trophic interactions among plants, pests, and natural enemies as the main driver of historical population dynamics [26]. Current research, however, has revealed substantial signals of environmental selection and adaptive evolution. Collectively, we propose that both tri-trophic relationships and environmental factors jointly shape the population dynamics of this species.

### 4.3. The Factors Driving Genetic Divergence

About the factors driving genetic divergence. Population structure analyses, including phylogenetic tree construction, Admixture modeling, PCA, and genetic differentiation (Fst), revealed that *P. japonica* consists of three genetic clades: a southern clade (G2) and two northern clades (G1 and G3), with some admixture among them. Additionally, demographic history analyses (Figure 2B,C) suggest that the divergence between the southern and northern populations occurred approximately 500 years ago. In the South China populations, selective sweep analysis identified high activity in energy metabolism, xenobiotic detoxification, and signal transduction pathways. Key functional genes include CYP450s, which mediate pesticide detoxification; CDAs, which are potential pesticide targets; and SYT1, which enhances adaptation to local environments. These genes play crucial roles in transcriptional and physiological responses to environmental stress, contributing to the adaptation of South China populations to challenging conditions.

Considering historical climate fluctuations and the differing pesticide application regimes across China, the observed “south–north” genetic divergence might primarily result from the adaptive evolution of the G2 clade in response to pesticide pressure, global warming, and geographical separation. Over the past century, climate warming has led to shifts in primary food [47]; for example, South China (e.g., southwestern, central, and southeastern regions) experienced a greater shift toward high-pesticide-demand crops (e.g., vegetables and orchards). The southwestern region saw the largest increase in high-dosage pesticide application, with southeastern provinces such as Guangdong, Fujian, and Hainan reporting extreme pesticide use intensities [48]. While pesticide application enhances crop yields, it also exerts strong selection pressure for insect resistance. Southern *P. japonica* populations (e.g., in Guangzhou, Fujian, Guangxi, and Yunnan) have developed resistance to common insecticides (abamectin, imidacloprid, beta-cypermethrin, and chlorpyrifos), ranging from moderate (10-fold) to high (50-fold) resistance levels. Continued pesticide application in these regions is expected to intensify this selection pressure, further enhancing resistance in *P. japonica* populations.

Heat stress responses also differ between northern and southern *P. japonica* populations. After pre-treatment, larvae from southern Guangdong exhibited significantly higher heat tolerance than those from northern Beijing, with fourth-instar larvae from Guangdong showing superior survival rates under heat stress [49]. The cumulative active temperature (≥10 °C) is substantially higher in South China than in North China. Previous studies indicate that South China experiences a higher frequency of extreme heat events and elevated mean summer surface air temperatures [50]. Several insect species in China have developed local thermal adaptations, with heat resistance decreasing with increasing latitude. For instance, South China populations of *Atractomorpha sinensis*, a dominant pest locust, exhibit greater heat tolerance than their northern counterparts [51]. In summary, prolonged exposure to high pesticide levels and frequent heat stress has driven the adaptive evolution of South China *P. japonica* populations, leading to significant genetic divergence from their northern counterparts.

These findings provide a roadmap for leveraging *P. japonica* more effectively: aiding the breeding of efficient commercial strains to combat greenhouse and climate challenges, and guiding a ‘regional precision release’ strategy that prioritizes the adaptive G2 strain in high-pressure, high-temperature environments like southern regions or greenhouses. In summary, by deciphering the adaptive mechanisms behind genetic divergence, we can transform evolutionary history into applicable strategies, paving the way for a paradigm shift in biocontrol from a ‘broad-spectrum and extensive’ approach to a ‘precise and efficient’ one.

## 5. Conclusions

To investigate the population evolutionary patterns and environmental adaptation basis of *P. japonica*, we performed whole-genome resequencing on 166 individuals from 29 populations to analyze population evolutionary history, genetic structure and genetic diversity, while identifying key genes involved in environmental adaptation. Population genetic analysis identified significant north–south divergence, largely attributed to adaptive evolution in the southern population to high temperatures and heavy insecticide use. phylogenetic analysis indicated that the northern population exhibits higher genetic diversity and an earlier origin than its southern counterpart, suggesting that the species originated in the Yellow River Basin of northern China. Demographic history and ecological modeling revealed a population bottleneck in *P. japonica* over the past 20,000 years due to harsh climate, especially during the LGM, followed by recent expansion driven by warming and its strong adaptive evolution capabilities. Genome-wide selective sweep analysis identified numerous key genes related to heat tolerance and insecticide resistance in the southern population, which are mainly enriched in pathways such as antioxidant activity, heme binding, phosphatase regulation, and signal transduction. Subsequent screening and functional validation of these candidates via RNAi and CRISPR are warranted to confirm their biological roles. The present study elucidated the genetic evolutionary patterns and environmental adaptation mechanisms of *P. japonica* by implementing a systematic nationwide population genomics survey and ecological niche modelling methods. This provides essential genetic resources for the directed breeding of highly adaptive *P. japonica* strains. This facilitates a critical transition in pest control from experience-based and reactive practices to predictive, precise, and preventive management, laying a solid groundwork for sustainable and eco-friendly agricultural pest control strategies.

## Data Availability

The original data presented in the study are openly available in the NCBI Bioproject database, https://www.ncbi.nlm.nih.gov/bioproject/ (accessed on 2 March 2026) under accession number PRJNA1227245, with the accession numbers of SRR32930111—SRR32930273, SAMN47375764—SAMN47375929. Detailed data information is shown in Table S12. Further inquiries can be directed to the corresponding authors.

## Supplementary Materials

The following supporting information can be downloaded at: https://www.mdpi.com/article/10.3390/biom16030421/s1. Figure S1: Variation of Cross-Validation (CV) error across different K values; Figure S2: The neighbor-joining (NJ) phylogenetic tree inferred using 166 adults; Figure S3: Linkage disequilibrium (LD) patterns of the *P. japonica* subgroup G1, G2 and G3; Figure S4: Functional enrichment of selected genes between G1 and G2 lineages (G1/G2) based on KOG databases; Figure S5: Functional enrichment of selected genes between G1 and G3 lineages (G1/G3) based on KOG database; Figure S6: Functional enrichment of selected genes between G1 and G3 lineages (G1/G3) based on KEGG database; Figure S7: Functional enrichment of selected genes between G2 and G3 lineages (G2/G3) based on KOG database; Figure S8: Functional enrichment of selected genes between G2 and G3 lineages (G2/G3) based on KEGG database. Table S1: The information of sample collection; Table S2: Genome total reads and mapping rates of 166 *Propylea japonica* samples; Table S3: The SNPs annotation information; Table S4: The indels annotation information; Table S5: The genetic parameters of each population/group; Table S6: The effective population sizes (Ne) and the number of migrants per generation (M) among *Propylea japonica* across different geographical regions; Table S7: The GO terms are selected for South China populations (G2) based on the top 1% threshold, (Fst and *θ*π, G1/G2); Table S8: The GO terms are selected for South China populations (G2) based on the top 5% threshold (Fst and *θ*π, G1/G2); Table S9: List and annotation of genes exhibiting genomic signatures of selection based on the top 1% threshold (Fst and *θ*π, G1/G2); Table S10: The GO terms are selected for North China populations (G3) based on the top 5% threshold (Fst and *θ*π, G1/G3); Table S11: The GO terms are selected for North China populations (G3) based on the top 5% threshold (Fst and *θ*π, G2/G3); Table S12: Information on data uploaded to the NCBI databases.
