# Supplementary material for: Genomic Insights into Local Adaptation and Evolutionary Trajectories of Propylea japonica
Source: Biomolecules. 2026 Mar 12;16(3):421. doi: 10.3390/biom16030421 (PMC13023841; doi:10.3390/biom16030421)
Supplement: Supplementary file 1 [file biomolecules-16-00421-s001.zip › biomolecules-4124465-Supplementary_Figures.pdf]

biomolecules

MDPI

Article

Genomic insights into local adaptation and evolutionary trajectories of *Propylea japonica*

Lijuan Zhang [1], Yan Shi [1], Mengqi Wang [2], Yang Xu [1], Xiaojie Yang [1], Man Zhao [1], Weizheng Li [1], Xianru Guo [1], Chenchen Zhao [1*] and Yuqiang Xi [1*]

[1] College of Plant Protection, Henan Agricultural University, Zhengzhou, 450046, China
[2] Society Work Department of the CPC Suifenhe Municipal Committee, Suifenhe, 157399, China

Table of Contents

Supplementary Figure S1 .... Error! Bookmark not defined.

Supplementary Figure S2 .... **2**

Supplementary Figure S3 .... Error! Bookmark not defined.

Supplementary Figure S4 .... Error! Bookmark not defined.

Supplementary Figure S5 .... Error! Bookmark not defined.

Supplementary Figure S6 .... Error! Bookmark not defined.

Supplementary Figure S7 .... **5**

Supplementary Figure S8 .... **5**

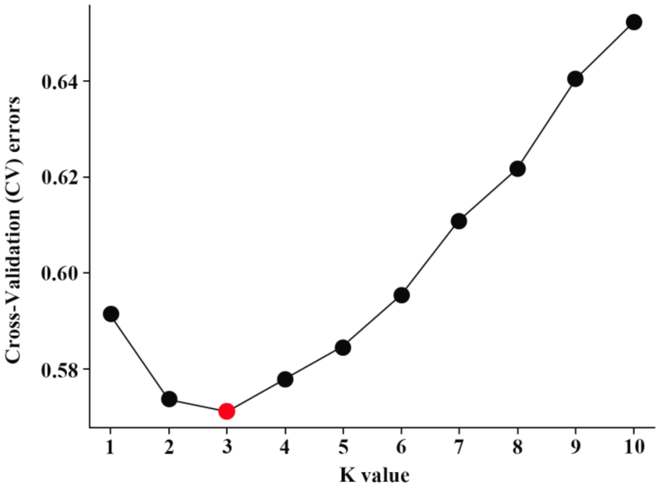

Supplementary Figure S1. Variation of Cross-Validation (CV) error across different K values. The x-axis represents the K value (1-10), and the y-axis denotes the CV error. The results indicate that the lowest error occurs at K=3, suggesting optimal model performance under this parameter.

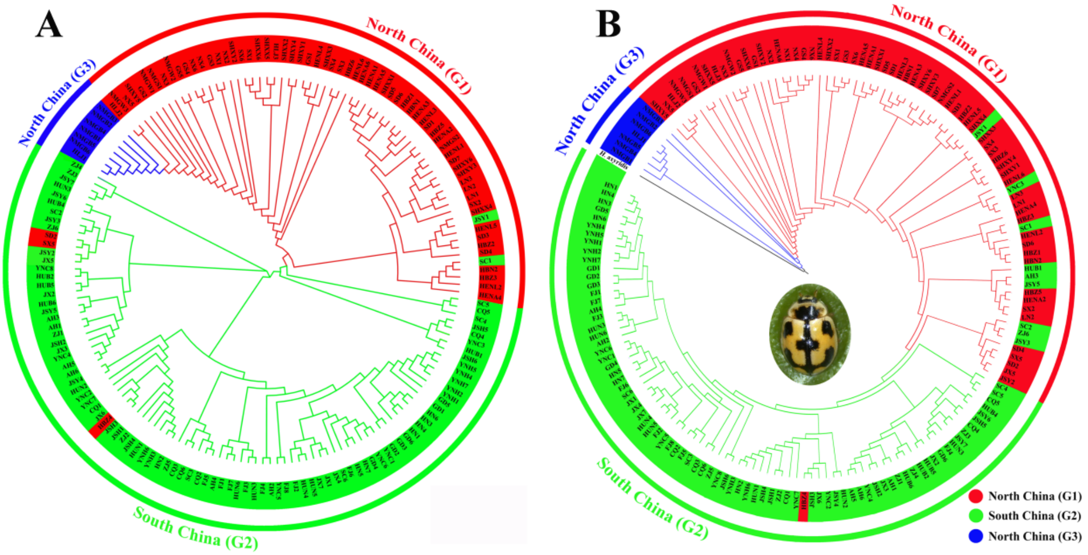

Supplementary Figure S2. The neighbor-joining phylogenetic tree inferred using 166 adults. **(A)** Unrooted NJ tree, (**B**) Rooted NJ tree.

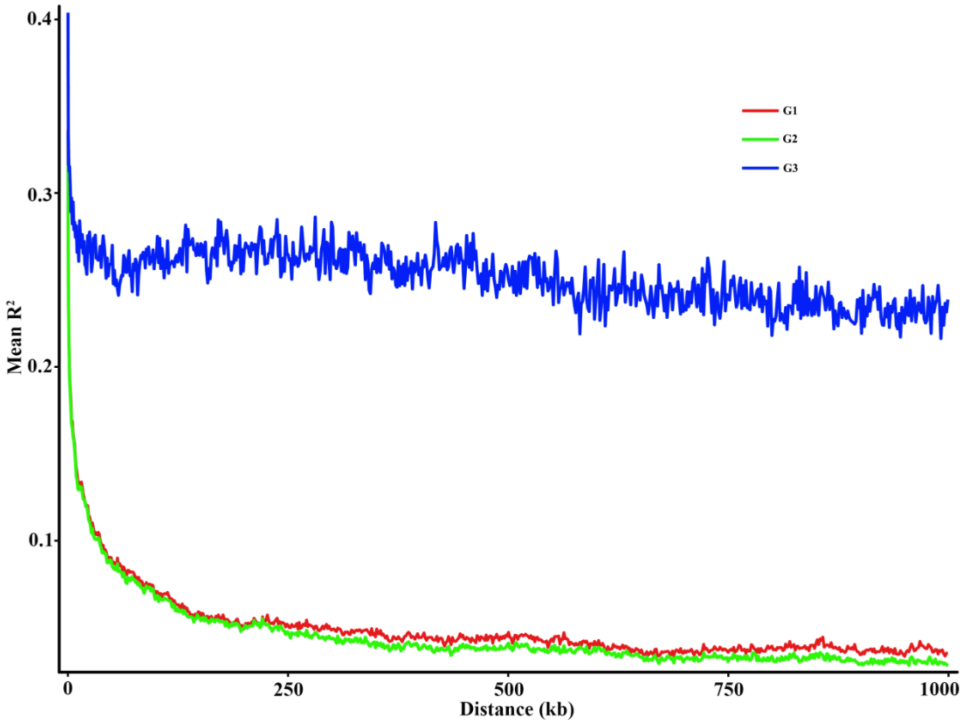

Supplementary Figure S3. Linkage disequilibrium (LD) patterns of the *P. japonica* subgroup G1, G2 and G3.

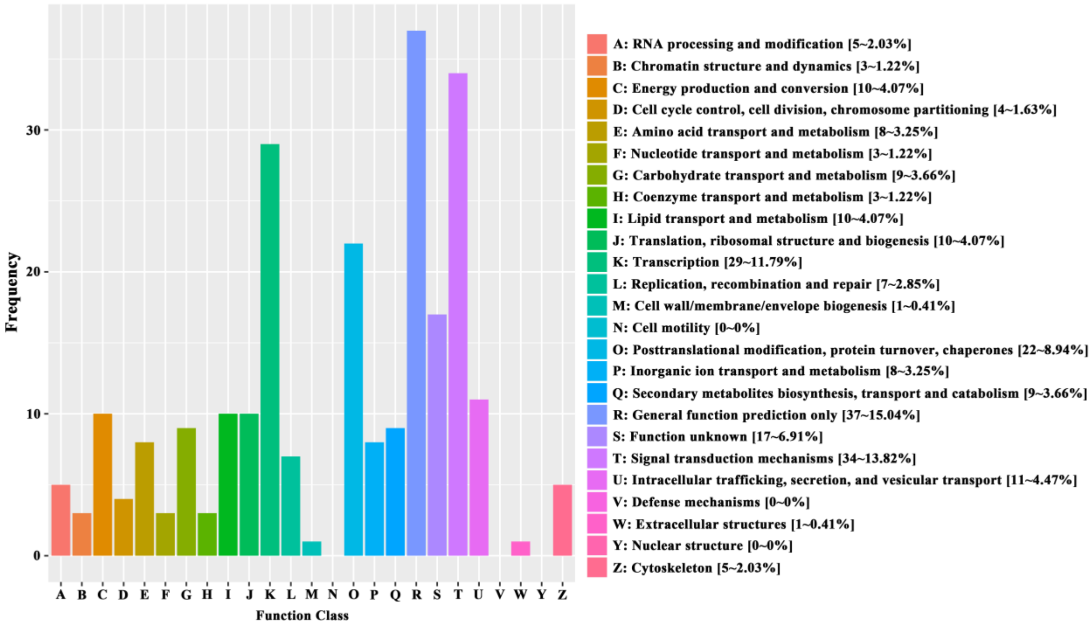

Supplementary Figure S4. Functional enrichment of selected genes between G1 and G2 lineages (G1/G2) based on KOG databases. KOG functional classification, categories [R] general function prediction (37–15.04%) and [T] signal transduction mechanisms (34–13.82%) showed the highest proportions, while categories such as [M] cell wall/membrane/envelope biogenesis (1–0.41%) were not significantly enriched. X axis indicates functional categories of genes; Y axis indicates the percentage of genes.

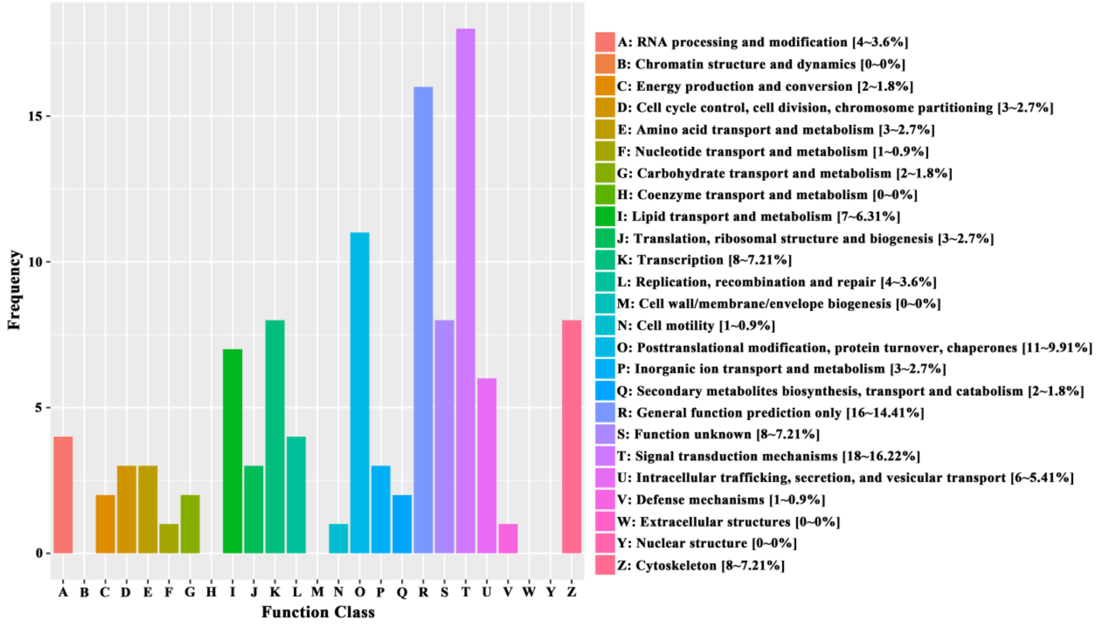

Supplementary Figure S5. Functional enrichment of selected genes between G1 and G3 lineages (G1/G3) based on KOG database. X axis indicates functional categories of genes; Y axis indicates the percentage of genes.

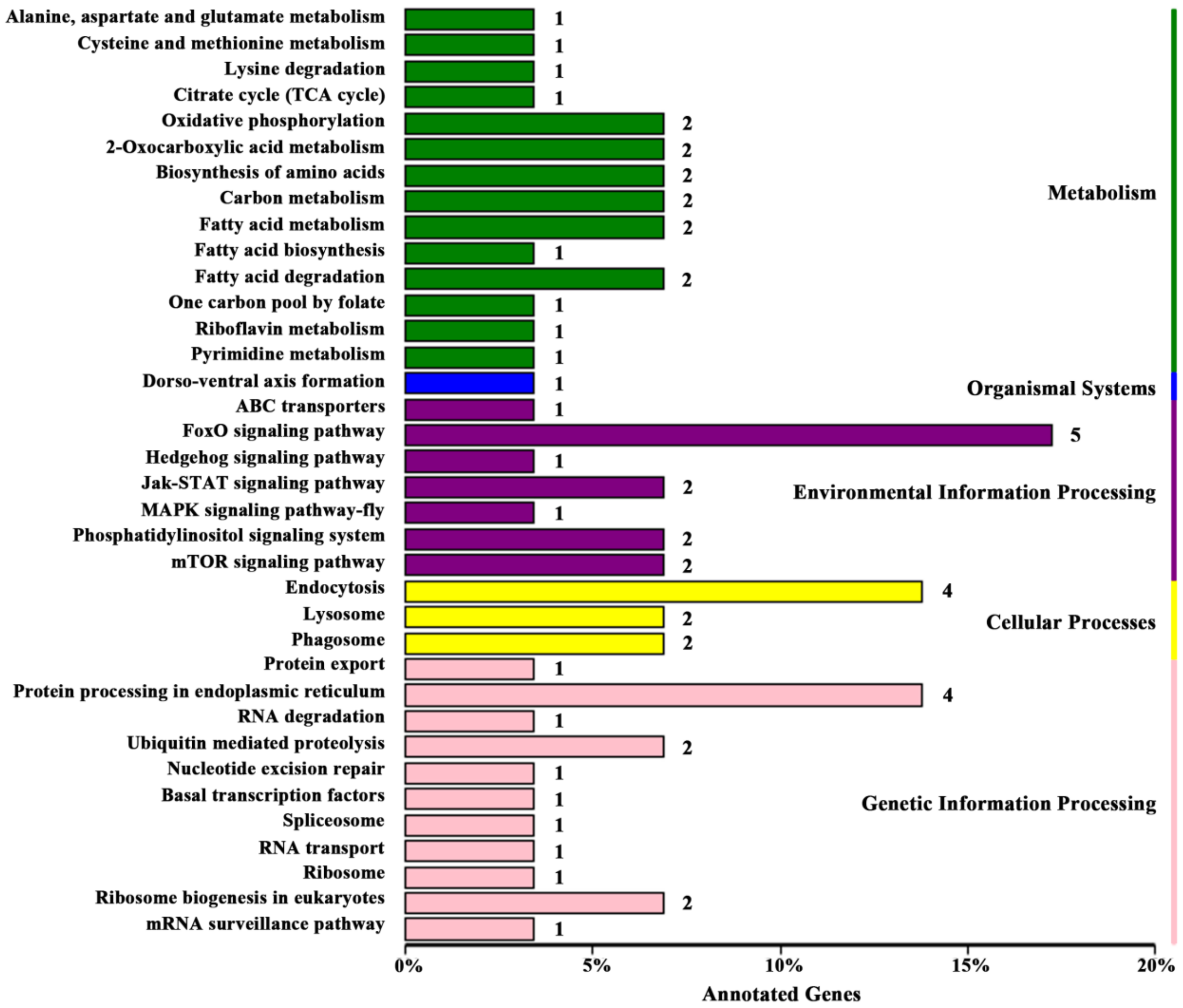

Supplementary Figure S6. Functional enrichment of selected genes between G1 and G3 lineages (G1/G3) based on KEGG database. The distribution of annotated genes within major biological categories, including cellular processes, metabolism, environmental information processing, genetic information processing, and organismal systems. Y-axis represents categories are grouped hierarchically; X-axis represents percentage of annotated genes per category; the numerical labels on the right side of the bar chart indicate the gene count.

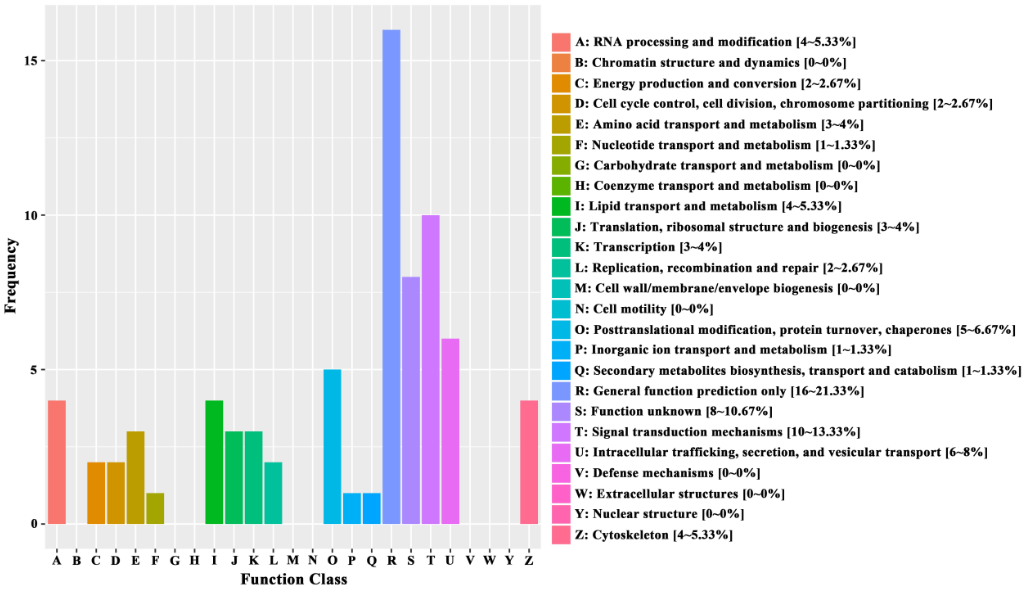

Supplementary Figure S7. Functional enrichment of selected genes between G2 and G3 lineages (G2/G3) based on KOG database. X axis indicates functional categories of genes; Y axis indicates the percentage of genes.

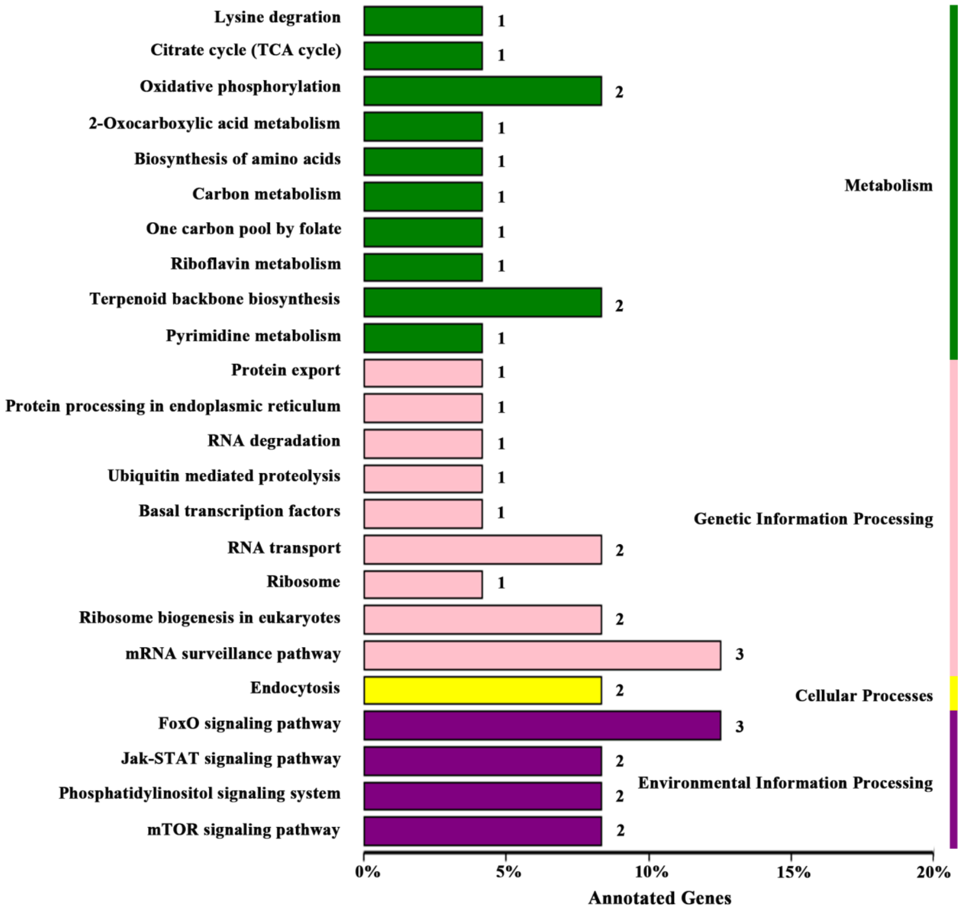

Supplementary Figure S8. Functional enrichment of selected genes between G2 and G3 lineages (G2/G3) based on KEGG database. The distribution of annotated genes within major biological categories, including cellular processes, metabolism, environmental information processing, genetic information processing, and organismal systems. Y-axis represents categories are grouped hierarchically; X-axis represents percentage of annotated genes per category; the numerical labels on the right side of the bar chart indicate the gene count.
